# Supplementary material for: Frontal cortex activity during the production of diverse social communication calls in marmoset monkeys
Source: Nat Commun. 2023 Oct 19;14:6634. doi: 10.1038/s41467-023-42052-5 (PMC10587070; doi:10.1038/s41467-023-42052-5)
Supplement: Supplementary file 1 — Supplementary Information [file 41467_2023_42052_MOESM1_ESM.pdf]

Supplementary Information for

Frontal cortex activity during the production of diverse social communication calls in marmoset monkeys

Lingyun Zhao*, Xiaoqin Wang*

*Correspondence to: lingyun.zhao@ucsf.edu, xiaoqin.wang@jhu.edu

This PDF file includes:

Supplementary Fig. 1

Supplementary Fig. 2

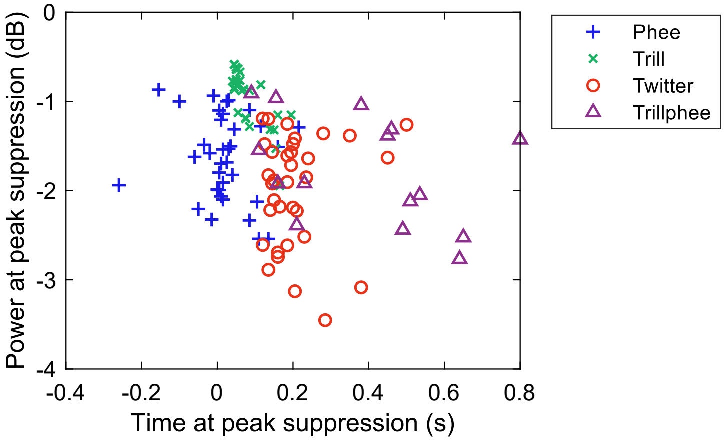

Supplementary Fig. 1 Scatter plot of the time and power at peak suppression of beta-band LFP for four call types. Each data point is from a recording site showing significant suppression for the call type indicated by color. One data point with the time at peak suppression greater than 0.8 sec is plotted on the border of the axes. Source data are provided as a Source Data file.

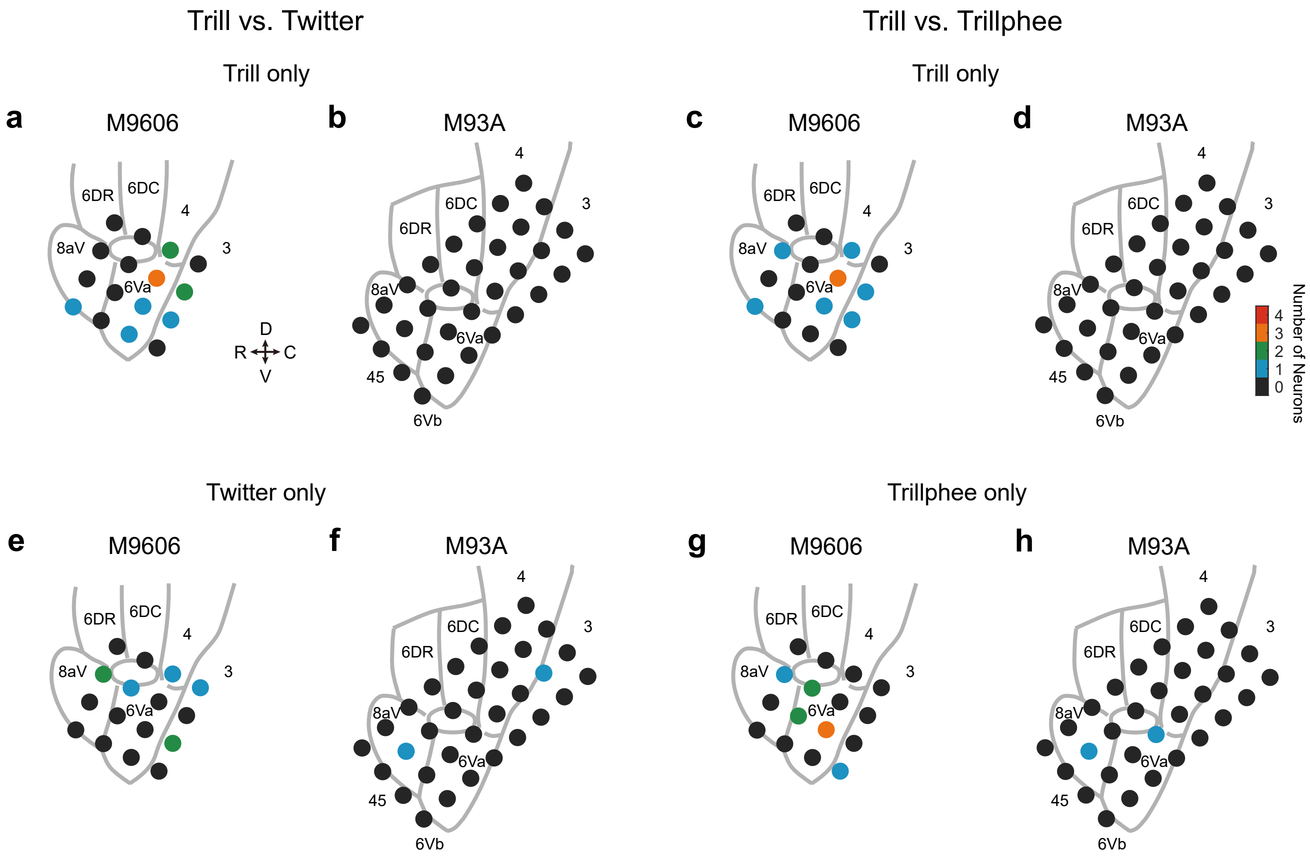

Supplementary Fig. 2 Spatial distribution of neurons showing modulation to only one call type when tested with two call types (same format as Fig. 8**i-l**). **a, b, e, f** Neurons tested with trill and twitter calls. **c, d, g, h** Neurons tested with trill and trillphee calls.
